# Academic Outcomes of School-Aged Children Born Preterm: A Systematic Review and Meta-analysis

**DOI:** 10.1001/jamanetworkopen.2020.2027

**Published:** 2020-04-03

**Authors:** Melinda McBryde, Grace C. Fitzallen, Helen G. Liley, H. Gerry Taylor, Samudragupta Bora

**Affiliations:** 1 Currently graduate students at School of Psychology, Faculty of Health and Behavioural Sciences, The University of Queensland, Brisbane, Australia; 2 Mothers, Babies and Women’s Health Program, Mater Research Institute, Faculty of Medicine, The University of Queensland, Brisbane, Australia; 3 Abigail Wexner Research Institute, Biobehavioral Health Centre, Nationwide Children’s Hospital, Columbus, Ohio; 4 Department of Pediatrics, The Ohio State University, Columbus

## Abstract

**Importance:**

Children born preterm are at an elevated risk of academic underachievement. However, the extent to which performance across domain-specific subskills in reading and mathematics is associated with preterm birth remains unclear.

**Objective:**

To conduct a systematic review and meta-analysis of academic outcomes of school-aged children born preterm, compared with children born at term, appraising evidence for higher- and lower-order subskills in reading and mathematics.

**Data Sources:**

PubMed/MEDLINE, PsycINFO, and the Cumulative Index of Nursing and Allied Health Literature electronic databases from January 1, 1980, to July 30, 2018, were searched for population, exposure, and outcome terms such as *child* (population), *preterm birth* (exposure), and education* (outcome).

**Study Selection:**

Peer-reviewed English-language publications that included preterm-born children and a comparison group of term-born children aged 5 to 18 years and born during or after 1980 and that reported outcomes on standardized assessments from cohort or cross-sectional studies were screened. Of the 9833 articles screened, 33 unique studies met the inclusion criteria.

**Data Extraction and Synthesis:**

Data were analyzed from August 1 to September 29, 2018. The Preferred Reporting Items for Systematic Reviews and Meta-analyses (PRISMA) guidelines were followed. Two reviewers independently screened the databases and extracted sample characteristics and outcomes scores. Pooled mean differences (MDs) were analyzed using random-effects models.

**Main Outcomes and Measures:**

Performance on standardized assessment of higher-order subskills of reading comprehension and applied mathematics problems; lower-order reading subskills of decoding, pseudoword decoding, and word identification; and lower-order mathematics subskills of knowledge, calculation, and fluency.

**Results:**

Outcomes data were extracted for 4006 preterm and 3317 term-born children, totaling 7323 participants from 33 unique studies. Relative to children born at term, children born preterm scored significantly lower in reading comprehension (mean difference [MD], −7.96; 95% CI, −12.15 to −3.76; *I*^2^ = 81%) and applied mathematical problems (MD, −11.41; 95% CI, −17.57 to −5.26; *I*^2^ = 91%) assessments. Across the assessments of lower-order skills, children born preterm scored significantly lower than their term-born peers in calculation (MD, −10.57; 95% CI, −15.62 to −5.52; *I*^2^ = 92%), decoding (MD, −10.18; 95% CI, −16.83 to −3.53; *I*^2^ = 71%), mathematical knowledge (MD, −9.88; 95% CI, −11.68 to −8.08; *I*^2^ = 62%), word identification (MD, −7.44; 95% CI, −9.08 to −5.80; *I*^2^ = 69%), and mathematical fluency (MD, −6.89; 95% CI, −13.54 to −0.23; *I*^2^ = 72%). The associations remained unchanged after sensitivity analyses for reducing heterogeneity.

**Conclusions and Relevance:**

These findings provide evidence that preterm birth is associated with academic underperformance in aggregate measures of reading and mathematics, as well as a variety of related subskills.

## Key Points

**Question:**

What is the extent of the associations between preterm birth and domain-specific subskills in reading and mathematics?

**Findings:**

This systematic review and meta-analysis of 33 unique studies comparing 4006 preterm and 3317 term-born children of school age across outcome domains showed moderate to large differences between the 2 groups. Preterm birth was associated with academic underperformance in aggregate measures of reading and mathematics, as well as a variety of related subskills.

**Meaning:**

This evidence suggests that children born preterm are at risk of significant academic difficulties in a variety of higher- and lower-order subskills in the reading and mathematics domains compared with term-born peers.

## Teaser

This systematic review and meta-analysis compares academic outcomes across higher- and lower-order subskills in reading and mathematics among school-aged children born preterm vs those born at term.

## Introduction

Rising worldwide rates of preterm birth (<37 weeks’ gestation) and the increasing survival of infants born prematurely are contributors to a global decrease in mean gestational age at birth.^1^ This trend makes the long-term developmental sequelae associated with this population a growing public health concern.^2^ A pressing issue for the families and future educators of this population is the risk posed by preterm birth for academic underachievement, one of the high-prevalence, low-severity impairments often associated with this population.^3^

Studies have focused on the academic domains of reading and mathematics. Reading deficits can have a cascading effect, in turn affecting academic performance in mathematics and the sciences.^4^ Reduced performance in mathematics may present far-reaching and perhaps lifelong repercussions. For example, the association between preterm birth and adult wealth is particularly mediated by low achievement in the mathematics domain during the formative school years.^5^ Despite the clear importance of reading and mathematics skills, the problem as it relates to the preterm population has been defined broadly, with little known of how component subskills are affected.^6^ Furthermore, educational professionals underappreciate the potential challenges faced by students with preterm birth histories and may be ill-prepared to address their needs.^7^ With a growing proportion of preterm births and an increasing emphasis on education in our workforces and economies, it is vital to identify and characterize these academic problems in school-aged children born preterm so that targeted interventions may be developed.

To date, studies report inconsistent findings regarding the nature and magnitude of difference in academic performance between preterm and term-born children.^8^ This variation may be a result of small sample sizes, diverse demographic characteristics, international diversity in educational curricula and standards, and methodological inconsistencies, including study design differences and variations in outcome measures. However, pertinent meta-analytic findings^8,9,10,11^ consistently reveal that children born preterm are at greater risk of academic challenges than their term-born peers in reading and mathematics and that mathematics deficits are most pronounced.

In a recent meta-analysis of reading performance at school age,^11^ children born preterm showed deficits in decoding and reading comprehension subskills compared with term-born children. Similarly, Aarnoudse-Moens et al^8^ found that very preterm children (<32 weeks’ gestation at birth) showed moderate to severe deficits in reading, spelling, and arithmetic. However, the applicability of these results is questionable because most of the samples included children born before antenatal corticosteroid and artificial surfactant treatments were routinely available for preterm children. Twilhaar and colleagues^9^ provided insight into the academic outcomes of preterm children born from 1990 onward. Findings from this more recent era were similar: 78% of very preterm children had special education needs and scored 0.44 and 0.52 SDs lower than term-born peers in reading and mathematics, respectively. Interestingly, results suggested that rates of academic deficits for preterm children have not improved. Most recently, a meta-analysis by Allotey and colleagues^10^ examined whether deficits in the preterm population persist throughout the academic career. Preterm children demonstrated lower reading and mathematics scores at primary school age, but decreased performance persisted through secondary school for reading only. This study used standardized measures, thus avoiding possible problematic comparisons of teacher-rated performance or special education needs, which may be inconsistent across study cohorts and more vulnerable to assessor bias. The authors found a gradient of increasing risk for mathematics deficits with decreasing gestational age. This association was not significant for reading outcomes.^10^

Against this background, the present study aimed to conduct a systematic review and meta-analysis to quantify the strength of association between preterm birth and performance in the reading and mathematics domains, with emphasis on profiling deficits in domain-specific subskills. A second aim was to characterize the nature of difficulties in reading and mathematics across the spectrum of prematurity. In addition, this study investigated how reading and mathematics problems present differently at various ages of assessment from 5 to 18 years in preterm and term-born children. Finally, this study aimed to determine the association between preterm birth and academic outcomes, comparing different birth eras as a means to identify possible temporal trends.

## Methods

### Study Selection

This systematic review and meta-analysis was conducted in accordance with the Preferred Reporting Items for Systematic Reviews and Meta-analyses (PRISMA) reporting guideline.^12^ The study’s PECO (population, exposure, comparator, and outcome) framework asked whether, among children of school age (population), preterm birth (exposure) compared with term birth (comparator) was associated with poorer academic outcomes (outcome). Inclusion criteria for studies were (1) a study sample consisting of preterm or low-birth-weight children aged 5 to 18 years and born during or after 1980; (2) use of a term-born comparison group; (3) academic outcomes assessed by validated, standardized tests and reporting mean (SD) scores; and (4) cohort or cross-sectional studies published in peer-reviewed journals in English. We searched PubMed/MEDLINE, PsycINFO, and the Cumulative Index of Nursing and Allied Health Literature electronic databases from January 1, 1980, to July 30, 2018, for title and abstract keywords relevant to the PECO question. A full list of search terms is available in eTable 1 in the Supplement. For studies that reported outcomes of the same cases (eg, at different ages), selection was determined by longest follow-up interval (ie, age at assessment). Where duplicate reports were found, only the study with the largest sample size was selected. Some articles (n = 7) shared cohorts but reported different outcomes (ie, scores from various subtests); sample independence was maintained in each quantitative synthesis.

### Outcomes Measures

The meta-analysis compared mean (SD) scores from standardized tests of reading and mathematics (and associated subskills). The demarcation of subskills came from investigating the content and structure of the psychoeducational measures, looking for commonalities, and categorizing the constructs they purport to measure. The assessment tools and corresponding constructs are displayed in eTable 2 in the Supplement. All assessment measures were normed to a mean (SD) of 100 (15) and were compared in this metric.

Reading domain skills were categorized using the following labels: overall reading, decoding (lower-order skill), and reading comprehension (higher-order skill). Decoding skills were further demarcated into word identification and pseudoword decoding, because the former can be automatic and achieved when a word is recognized, whereas the latter relies on the application of phonetic rules.

Mathematical skills were categorized into constructs of overall mathematics, mathematical knowledge, calculation, mathematical fluency, and applied problems. Subtests used to assess what the authors have termed mathematical knowledge require children to perform a range of tasks, including counting, recognizing numerals and mathematical symbols, and arriving at correct answers to simple addition, subtraction, and multiplication problems (often rote learned and automatically recalled). Calculation differs from mathematical knowledge in that children must perform mathematical computations either mentally (overlapping somewhat with mathematical knowledge) or with paper and pencil. Both mathematical knowledge and calculation skills are fundamental to more complex tasks of mathematical reasoning. Mathematical fluency was assessed with measures that included a time factor and evaluated the child’s ability to efficiently recall mathematical facts or conduct computations. The highest-order mathematical skill defined was applied problems, which require multiple processes, namely, that the child readily interprets the problem, identifies a suitable mathematical approach to solving it, and accurately arrives at an answer.

### Study Quality

Studies retained at the final review phase were assessed using the Newcastle-Ottawa Quality Assessment Scale.^13^ This scale, developed to assess the overall methodological quality of nonrandomized studies and potential risk of bias, uses a 9-point classification. Studies were scored from 0 to 9, with 0 indicating low quality and high risk of bias, and 9 indicating high quality and low risk of bias.

### Statistical Analysis

Data were analyzed from August 1 to September 29, 2018. Preterm and term-born children’s mean differences (MDs) in outcome scores, extracted from the various eligible studies, were compared using the Cochrane Review Manager (RevMan 5) software, version 5.3.5, with the inverse-variance weighting method and random-effects models. The mean scores were used, and not standardized, because all the psychoeducational measures drawn from the studies had a mean of 100 and SD of 15. As detailed earlier, 5 meta-analytic comparisons were made for the reading domain and 5 for mathematics. Pooled effect sizes were calculated for each comparison, indicating the direction and magnitude of the exposure effects (preterm vs term birth). The upper and lower limits for the 95% CIs correspond with the conventional 5% significance level used in hypothesis testing. Heterogeneity of effect sizes was quantified using the *I*^2^ statistic, where a value of approximately 25% constitutes low heterogeneity; 50%, moderate heterogeneity; and 75%, high heterogeneity.^14^

Three further types of comparisons were conducted. First, the dose effect of exposure to prematurity was examined by comparing studies’ outcomes by mean gestational age of their preterm group. For this analysis, gestational category was determined using studies that reported mean gestational age at birth. If studies also reported birth weight, the reviewers (M.M. and S.B.) ensured that this was an expected value based on the degree of prematurity (or reported term birth for comparison groups). Where studies reported only birth weights, gestation was inferred according to published guidelines.^15^ Where ranges crossed gestational categories, means were used to categorize the samples. Second, studies were categorized by age at assessment to determine whether these skills are differentially affected at various periods throughout the academic career. Third, the data were compared by era of birth, to examine whether cohort effects may also have an association with reading and mathematics deficits. Three birth eras were defined: 1980 to 1990, 1991 to 2000, and 2001 to 2018. Where cohorts’ recruitment years crossed these dates of classification (n = 3) and could not be neatly categorized, the first reported year of recruitment was used to assign the entire cohort. To obtain the largest possible sample sizes for these 3 types of contrasts and increase the precision of the effect size estimates, dependent variables included aggregate measures of achievement, followed by subtests of higher-order skills, and then subtests of lower-order skills. Results from the same participants were not repeated more than once in each contrast (or the subgroups they constituted). Jackknife sensitivity analysis, in which 1 study at a time is removed from the comparison, was performed to ascertain whether a particular sample accounted for observed effects (ie, whether the significance of the original finding changed with 1 study’s exclusion). This sensitivity analysis was performed for the aggregate measures of reading and mathematics as well as associated subskills.

## Results

### Search Results

The steps of the article screening processes are illustrated in the PRISMA flowchart (eFigure 1 in the Supplement). Primary database searches produced 51 062 articles. Another 25 articles were identified by the secondary reviewer (G.C.F.) and perusal of reference lists from relevant meta-analyses. After removing duplicates, 9833 articles were screened; following inclusion criteria, a total of 33 unique studies^16,17,18,19,20,21,22,23,24,25,26,27,28,29,30,31,32,33,34,35,36,37,38,39,40,41,42,43,44,45,46,47,48^ were eligible for inclusion in the meta-analysis.

### Study Characteristics

The characteristics of the 33 included studies are shown in the Table. The study samples were derived from the United States, Canada, the United Kingdom, Ireland, Australia, New Zealand, and India. Participants were assessed at 5 to 18 years of age. Sample sizes of preterm children ranged from 10 and 298; of term-born children, from 10 to 262. Across all studies, 4006 preterm and 3317 term-born children were included among the 7323 unique participants. The earliest preterm participants were born in 1980 to 1981 and the most recent preterm participants included in the syntheses were born in 2005.

**Table.** Study Characteristics

Source	Country of birth	Year of birth	Age at assessment, y	Preterm group					Term-born comparison group				NOS score
				Exposure^a^	Gestational age, mean (SD), wk	Birth weight, mean (SD), g	Sample size, No.	Male, %	Gestational age, mean (SD), wk	Birth weight, mean (SD), g	Sample size, No.	Male, %	
Anderson et al,^21^ 2003	Australia	1991-1992	8	VPT, ELBW	NR	NR	298	46.5	NR	>2500^b^	262	46.6	9
Andreias et al,^22^ 2010	United States	1992-1995	8	ELBW	26.4 (2.0)	810 (124)	183	38	≥37^b^	3300 (513)	176	37	9
Assel et al,^23^ 2003	United States	1990-1992	8	PT	29.7 (2.5)	1111 (264)	160	54	39.9 (0.2)	3212 (735)	90	45	7
Botting et al, 1998^24^	United Kingdom	1980-1981	12	VLBW	≤30^b^	<1501^b^	138	NR	NR	NR	163	NR	8
Bowen et al,^25^ 2002	Australia	1985-1990	8	EPT or ELBW	27.2 (2.0)	893 (133)	82	58	39.4 (1.3)	3464 (542)	48	58	8
Brumbaugh et al,^26^ 2016	United States	2000-2006	9-10	LPT	NR	2700	52	55.8	>37	3590	74	50	8
Chaudhari et al,^27^ 2004	India	1987-1989	12	LBW, VLBW	NR	1549.0 (242.3)	180	68	NR	>2500	90	63	7
Cheong et al,^19^ 2017	Australia	1997	8	EPT	25.6 (1.2)	820 (173)	133	56	>37	≥2500	168	NR	9
Cheong et al,^19^ 2017	Australia	2005	8	EPT	25.8 (1.2)	867 (193)	140	49	>37	≥2500	189	NR	9
Downie et al,^28^ 2005	Canada	1984-1987	11	EPT, ELBW	26	814	39	NR	40.6	NR	15	NR	7
Doyle et al,^29^ 2000	Australia	1991-1992	18	EPT, ELBW	26.7 (1.9)	NR	298	46	39.2 (1.4)	NR	262	48	8
Frye et al,^16^ 2009	United States	1991-1992	12	PT	31.2 (0.7)	NR	94	50	40.0 (0.0)	3491 (110)	97	57.1	8
Frye et al,^16^ 2009	United States	1991-1992	12	PT	29.7 (1.2)	907 (75)	62	50	40.0 (0.0)	3491 (110)	97	57.1	8
Gross et al,^30^ 2001	United States	1985-1986	10	VPT, EPT	28.3 (2.2)	1147.0 (337.3)	118	NR	NR	NR	119	NR	8
Grunau et al,^31^ 2002	Canada	1982-1987	9	ELBW	26.0	718.8	74	NR	40.0	3540	30	NR	8
Grunau et al,^32^ 2004	Canada	1981-1986	17	ELBW	25.8	719	53	32	40	3506	31	50	6
Hutchinson et al,^33^ 2013	Australia	1997	8	EPT, ELBW	26.5 (2.0)	833 (164)	189	52.9	39.3 (1.1)	3506 (1455)	173	53.2	9
Johnson et al,^34^ 2011	United Kingdom and Ireland	1995	11	EPT	24.5 (0.7)	745 (130)	219	46.1	NR	NR	153	41.8	7
Lee et al,^35^ 2011	United States	1991-2001	9-16	PT	28.8 (2.7)	1215 (465)	65	53.8	39.5 (1.2)	3425 (499)	35	45.7	8
Litt et al,^18^ 2012	United States	1992-1995	14	ELBW	26.4 (2.0)	815 (124)	181	39	NR	3260 (524)	115	36	8
Loe et al,^36^ 2012	United States	1991-2001	9-16	PT, LBW	29.8 (2.7)	1226 (446)	72	47	39.7 (1.2)	3474 (492)	42	48	8
McGrath and Sullivan,^37^ 2002	United States	1985-1989	8	PT	31.9 (1.9)	1618.7 (83.6)	48	50	39.9 (0.9)	3399.8 (358.2)	37	50	8
Northam et al,^38^ 2012	United Kingdom	1989-1994	13-18	PT	27 (2.0)	1081 (385)	50	NR	NR	NR	30	NR	7
Pritchard et al,^39^ 2009	New Zealand	1998-2006	6	EPT, VPT	27.9 (2.3)	1071 (315)	102	52	39.5 (1.2)	3575 (410)	108	54.6	8
Rickards et al,^40^ 2001	Australia	1980-1982	14	VLBW	29.3 (2.0)	1167 (215)	120	54.2	39.9 (1.0)	3417 (432)	41	61	8
Rose et al,^41^ 2011	United States	1995-1997	11	PT, LBW	29.7 (2.8)	1165.2 (268.4)	44	56.8	38-42^b^	>2500^b^	87	48.3	8
Sayeur et al,^42^ 2015	Canada	2006	7-8	EPT, VPT	28.7 (1.8)	1222. (238.2)	10	50	38.7 (0.9)	3329.4 (539.4)	10	60	6
Short et al,^43^ 2003	United States	1989-1991	8	VLBW	30.0 (2.0)	125 (176)	75	55	40.0 (1.0)	3451 (547)	99	49	9
Simms et al,^20^ 2015	United Kingdom	2001-2003	8-10	VPT	28.6 (2.0)	1213.2 (365.4)	115	54.8	NR	NR	77	51.9	9
Tandon et al,^44^ 2000	India	1985-1989	5-9	LBW	36.2 (2.9)	181 (248)	27	43.5	39.6 (1.2)	2850 (363)	28	66	7
Tandon et al,^44^ 2000	India	1980-1985	9-13	LBW	36.0 (2.5)	1740 (195)	32	52.6	39.8 (1.3)	2850 (331)	29	56	7
Taylor et al,^45^ 1995	United States	1982-1986	6-7	ELBW	NR	660 (77)	35	28.6	NR	3341 (635)	58	36.2	8
Taylor et al,^46^ 2008	United States	1990-1992	8	VLBW	33.3 (5.4)	1857.8 (1128.0)	155	NR	NR	NR	82	NR	8
Taylor et al,^47^ 2011	United States	2001-2003	5-6	EPT	25.9 (1.6)	818 (174)	148	45.9	>36^b^	3382 (446)	111	45.9	8
Taylor et al,^48^ 2016	Australia	2001-2003	7	VPT	27.5 (1.9)	962 (223)	194	53	39.1 (1.3)	3323 (508)	70	49	8
Woodward et al,^17^ 2017	New Zealand	1998-2000	9	VPT	27.8 (2.4)	1054.4 (313.8)	100	51	39.5 (1.2)	3580.3 (414.5)	107	54.3	9

Abbreviations: ELBW, extremely low birth weight; EPT, extremely preterm; LBW, low birth weight; LPT, late preterm; NOS, Newcastle-Ottawa Quality Assessment Scale; NR, not reported; PT, preterm; VLBW, very low birth weight; VPT, very preterm.

^a^ In the preterm group, ELBW indicates less than 1000 g; LBW, less than 2500 g; VLBW, 1000 to 1500 g; EPT, less than 28 weeks’ gestation; LPT, 32 to 36 weeks’ gestation; PT, less than 37 weeks’ gestation; and VPT, 28 to 32 weeks’ gestation.

^b^ Denotes inclusion criteria for studies where means and SDs were not provided.

### Study Quality Assessment

Scores on the Newcastle-Ottawa Quality Assessment Scale for the 33 final studies ranged from 6 (indicative of fair quality) to 9 (the highest rating possible) (Table). The median score was 8, indicating that most studies were of good quality and showed low risk of bias. Studies received lower ratings in 3 areas: comparability of cohorts with the study design or analysis not controlling for socioeconomic status or another variable (n = 10), comparison group not drawn from the same community as the preterm cohort or description inadequate (n = 10), and inadequacy of follow-up of cohorts where attrition exceeded 15% or no statement was provided regarding follow-up (n = 14).

### Preterm Birth and Academic Outcomes Profile

**Figure 1.**
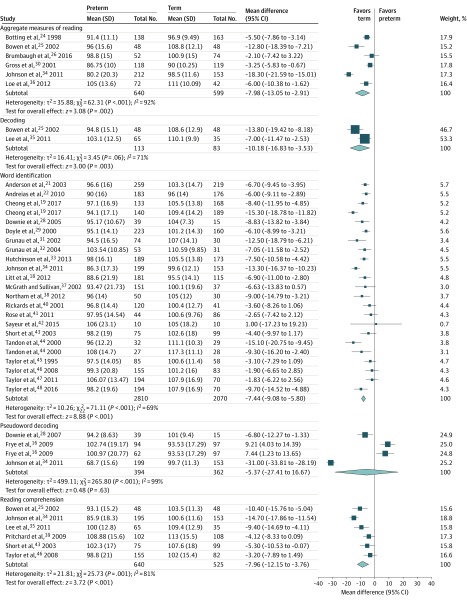
Inverse-Variance Random-Effects Forest Plot of Reading Domain, Including Subskills, for Preterm and Term-Born Children

The meta-analysis found preterm children are at risk of significant academic difficulties in aggregate measures of reading (MD, −7.98; 95% CI, −13.05 to −2.91; *I*^2^ = 92%) and aggregate measures of mathematics (MD, −12.90; 95% CI, −23.38 to −2.43; *I*^2^ = 97%) as well as a variety of related subskills, such as mathematical knowledge (MD, −9.88; 95% CI, −11.68 to −8.08; *I*^2^ = 62%) and calculation (MD, −10.57; 95% CI, −15.62 to −5.52; *I*^2^ = 92%), compared with term-born peers. Children born preterm underperformed relative to those born at term in the higher-order skill of reading comprehension (MD, −7.96; 95% CI, −12.15 to −3.76; *I*^2^ = 81%) as well as the lower-order reading skills of decoding (MD, −10.18; 95% CI, −16.83 to −3.53; *I*^2^ = 71%) and word identification (MD, −7.44; 95% CI, −9.08 to −5.80; *I*^2^ = 69%) (Figure 1). Preterm and term-born children did not differ in terms of pseudoword decoding performance (MD, −5.37; 95% CI, −27.41 to 16.67; *I*^2^ = 99%). However, the 2 independent samples of Frye and colleagues^16^ are atypical. In contrast with the existing literature, and possibly associated with sample characteristics, the preterm children outperformed term-born peers on the Woodcock Johnson Test of Achievement Word Attack measure,^49^ and the comparison group scored more than 6 points below the standardized test’s normed mean of 100. When these outlying samples were excluded, preterm children scored significantly worse than term-born counterparts (MD, −19.02; 95% CI, −42.73 to −4.70; *I*^2^ = 98%), indicating that the original finding of null difference should be interpreted with caution.

**Figure 2.**
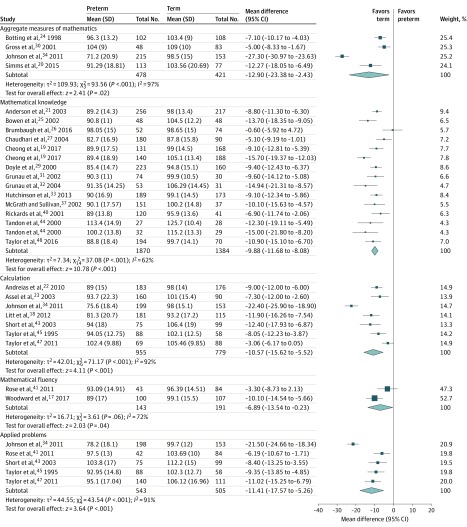
Inverse-Variance Random-Effects Forest Plot of Mathematics Domain, Including Subskills, for Preterm and Term-Born Children

Preterm children had deficits in all mathematics subskills compared with term-born peers (Figure 2). The least pronounced deficit appeared to be mathematical fluency (MD, −6.89; 95% CI, −13.54 to −0.23; *I*^2^ = 72%). However, the relatively small sample sizes for this comparison (aggregated samples of 143 preterm and 191 term-born participants) reduces the certainty of this estimate. Differences in mean scores from 543 preterm and 505 term-born participants suggest that children born preterm had significant deficits in the higher-order subskill of applied problems (MD, −11.41; 95% CI, −17.57 to −5.26; *I*^2^ = 91%).

Because of the high heterogeneity present in these contrasts, and to address potential bias caused by outlying data, a jackknife sensitivity analysis was undertaken to examine whether any particular study had a significant effect on the pooled effect size by removing 1 sample at a time. Results can be found in eTable 3 in the Supplement. Heterogeneity was reduced to lower or moderate levels, and the reported results remain unchanged in terms of directionality and significance.

### Preterm Birth and Academic Outcomes Profile Stratified by Gestational Age at Birth

Extremely preterm children born at less than 28 weeks’ gestation (aggregated sample of 2460) had significantly impaired reading performance compared with term-born peers (aggregated sample of 1955) (MD, −8.54; 95% CI, −10.52 − 6.55; *I*^2^ = 79%) (eFigure 2 in the Supplement). Conversely, very preterm children born at 28 to 32 weeks’ gestation (n = 802) did not exhibit later reading deficits compared with term-born children (n = 710) (MD, −1.42; 95% CI, −4.58 to 1.75; *I*^2^ = 73%). Again, the samples from Frye and colleagues,^16^ with outlying mean reading scores, may distort the overall results. After excluding this study, very preterm children exhibited comparative deficits (MD, −3.80; 95% CI, −5.41 to −2.20; *I*^2^ = 0%). Late-preterm children born at 33 to 37 weeks’ gestation (n = 162) showed marked reading deficits compared with term-born children (n = 168) in the final subgroup analysis (MD, −8.07; 95% CI, −14.29 to −1.84; *I*^2^ = 73%).

Mathematics impairments were more pronounced than reading deficits for children born within the same extremely preterm category of less than 28 weeks’ gestation (MD, −11.92; 95% CI, −14.60 to −9.24; *I*^2^ = 85%) (eFigure 3 in the Supplement). For an aggregated sample of 710 very preterm children born from 28 to 32 weeks’ gestation, significant mathematics impairments were also apparent when compared with an aggregated sample of 618 term-born peers (MD, −7.60; 95% CI, −9.25 to −5.96; *I*^2^ = 1%). For a sample of 342 children born at 33 to 37 weeks’ gestation compared with 258 term-born peers, effect sizes remained large (MD, −7.98; 95% CI, −12.81 to −3.16; *I*^2^ = 72%).

### Preterm Birth and Academic Outcomes Profile Stratified by Assessment Age

We defined 3 age subgroups: 5 to 8 years, 9 to 11 years, and 12 to 18 years. In reading ability, preterm children aged 5 to 8 years performed significantly worse than term-born counterparts (MD, −7.38; 95% CI, −9.69 to −5.07; *I*^2^ = 69%), as did those aged 9 to 11 years (MD, −8.93; 95% CI, −14.42 to −3.43; *I*^2^ = 91%) (eFigure 4 in the Supplement). Preterm reading deficits were significant but less pronounced when children were assessed at 12 to 18 years of age, with samples of 993 preterm and 776 term-born children (MD, −3.35; 95% CI, −6.70 to −0.01; *I*^2^ = 83%). With the potentially problematic outlying samples of Frye and colleagues^16^ excluded, preterm reading deficits remained significant but less pronounced in this group aged 12 to 18 years (MD, −6.01; 95% CI, −7.38 to −4.65; *I*^2^ = 0%). In contrast, the magnitude of deficits in mathematics in preterm groups was similar across age groups (eFigure 5 in the Supplement).

### Preterm Birth and Academic Outcomes Profile Stratified by Different Birth Eras

Comparing subgroup effect sizes revealed that, although reading deficits were most pronounced in those born from 1980 to 1990 (MD, −7.34; 95% CI, −9.38 to −5.30; *I*^2^ = 60%), these deficits continue to be evident in preterm children who may have received advanced neonatal care from 1991 to 2000 (MD, −4.58; 95% CI, −8.18 to −0.97; *I*^2^ = 90%) and from 2001 to 2018 (MD, −7.89; 95% CI, −15.46 to −0.32; *I*^2^ = 87%) (eFigure 6 in the Supplement). In the mathematics domain, the most severe impairments were found in cohorts of preterm children born during or after 2001 (eFigure 7 in the Supplement). When we compared 587 preterm children with 446 term-born counterparts during this period, mathematics impairments for preterm children were pronounced (MD, −12.68; 95% CI, −15.16 to −10.21; *I*^2^ = 23%).

## Discussion

Our findings showed that children born preterm, relative to term-born peers, had significant deficits in aggregate measures of the domains of reading and mathematics (as assessed on standardized achievement tests). These deficits appear more pronounced in the mathematics domain and the associated subskills of mathematical knowledge, calculation, and applied problems than in the reading domain and its subskills. However, these findings are implied only by pooled effect sizes of greater magnitude for mathematics and not investigated statistically because of problems involving sample independence. These results align with those from previous meta-analyses^8,9,10^ and suggest particular deficits in mathematics relative to reading. In the reading domain, lower-order (ie, decoding and word identification) and higher-order (ie, reading comprehension) subskills appear to be associated with preterm birth somewhat equally. This same finding was reported by Kovachy and colleagues^11^ in their meta-analysis of reading abilities in preterm children.

Among the effect size magnitudes of mathematics subskills, the higher-order skill of applied problems appears most associated with preterm birth, compared with lower-order abilities such as mathematical knowledge and calculation. This novel finding may be associated with working memory, a critical factor in mathematical success, because applied problems require children to derive and process information about the mathematical problem while simultaneously retrieving contextual information from long-term memory. The finding of a deficit in mathematics fluency in preterm children is also revealing; it has been suggested that this subskill is particularly important to performing well in school assessments.^17^

Although pooled effect sizes suggest pronounced deficits in extremely preterm children, late-preterm children also showed greater mean score differences in reading and mathematics than those born very preterm. This finding suggests that all preterm children are at risk of academic underperformance, not only those born at the lower bounds of gestational age. In exploring possible differential effects in associations between preterm birth and age at assessment, the present study presented an arguably more fine-grained and useful demarcation of age groups than the meta-analysis of Allotey and colleagues^10^ and included more studies and larger sample sizes. Results suggest that preterm children show significant deficits in reading at all ages of assessment (5-18 years), but that the MDs in scores are reduced somewhat relative to term-born children in later school years. Results are consistent with longitudinal studies of developmental changes in reading in preterm samples^18,50^ and raise the possibility that preterm children develop adaptive strategies in this domain. The findings also suggest, in contrast, that preterm-born youths face deficits in mathematics from early schooling to high school, with large and persisting disparities remaining between these children and their term-born peers.

Although deficits in reading were most pronounced in preterm children born during an earlier era of neonatal intensive care (ie, 1980-1990), performance gaps were found between these children and term-born peers across eras in reading and mathematics. These findings align with those of previous studies involving meta-regressions.^9,51^ There appears to be a substantial achievement gap between children born preterm and their term-born peers in mathematics in the most recent era (ie, 2001-2018). The reasons for substantial and possibly increasing academic difficulties among more recent preterm cohorts is unclear but consistent with findings from a population-based study.^19^ These findings have implications in a global job market that increasingly demands mathematical competence and in light of research suggesting that sound mathematical skills protect preterm children from decreased earning potential as adults.^5^

Differences in the etiology and presentations of learning difficulties in preterm compared with term-born groups suggest the need for measures that screen for problems specific to the preterm population.^20,52^ This comprehensive meta-analysis is the first, to our knowledge, to delineate academic subskills and their associations with preterm birth, and findings of this type will inform efforts to develop appropriate screening measures. Findings may also prove useful to teachers and education specialists in developing targeted interventions or specialized teaching plans for students born preterm who experience academic problems.

### Limitations

A key limitation of this study is the high heterogeneity present in many of the comparisons, suggesting that nonrandom factors (eg, changes in neonatal care practices, changes to educational curricula) and likely moderating factors influenced the generated effect sizes. This issue may limit the precision with which the results estimate true effects and the applicability of our findings to children born preterm in today’s educational system. However, this study partially addressed this problem by performing sensitivity analysis for some comparisons and found no changes in results despite reductions in heterogeneity. This process provides evidence of outcome specificity, that is, true associations between preterm birth and the academic deficits discussed. Another limitation of this study is that the meta-analysis relied on mean scores from standardized tests. Psychoeducational batteries are normed to the general population and may not be sensitive to the patterns of learning deficits in children born preterm. Another potential problem of this study is the use of low birth weight as a proxy for preterm birth in 36% (12 of 33) of included studies. These low-birth-weight samples possibly included children born small for gestational age, thus introducing a confounding variable. In addition, a search for gray literature was not conducted to complement the comprehensive database searches. However, because the resources required to conduct prospective cohort studies place constraints on the number of such investigations, the applied search strategy likely identified relevant research. Publication lists of major cohort studies in this field were also perused to ensure that no main sources of data were overlooked.

## Conclusions

Although the present study provides a comprehensive examination of the association between preterm birth and academic achievement, further meta-analyses are needed to investigate potential mediating and moderating factors such as socioeconomic status and comorbid medical and behavioral problems. The development of measures that are more sensitive to reading and mathematics deficits in preterm children than traditional psychoeducational batteries may also clarify the nature of academic deficits in children born preterm.

## Notes

Supplement. **eTable 1.** List of Keywords Used in Database Searches **eTable 2.** List of Assessment Measures from Included Studies **eTable 3.** Jackknife Sensitivity Analysis for Academic Subskills Comparisons **eFigure 1.** PRISMA Flowchart of Study Selection Process **eFigure 2.** Inverse-Variance Random-Effects Forest Plot of Associations Between Gestational Age and Reading Outcomes **eFigure 3.** Inverse-Variance Random-Effects Forest Plot of Associations Between Gestational Age and Mathematics Outcomes **eFigure 4.** Inverse-Variance Random-Effects Forest Plot of Associations Between Assessment Age and Reading Outcomes **eFigure 5.** Inverse-Variance Random-Effects Forest Plot of Associations Between Assessment Age and Mathematics Outcomes **eFigure 6.** Inverse-Variance Random-Effects Forest Plot of Associations Between Birth Era and Reading Outcomes **eFigure 7.** Inverse-Variance Random-Effects Forest Plot of Associations Between Birth Era and Mathematics Outcomes
