# Supplementary material for: Academic Outcomes of School-Aged Children Born Preterm: A Systematic Review and Meta-analysis
Source: JAMA Netw Open. 2020 Apr 3;3(4):e202027. doi: 10.1001/jamanetworkopen.2020.2027 (PMC7125435; doi:10.1001/jamanetworkopen.2020.2027)
Supplement: Supplement. — eTable 1. List of Keywords Used in Database Searches eTable 2. List of Assessment Measures from Included Studies eTable 3. Jackknife Sensitivity Analysis for Academic Subskills Comparisons eFigure 1. PRISMA Flowchart of Study Selection Process eFigure 2. Inverse-Variance Random-Effects Forest Plot of Associations Between Gestational Age and Reading Outcomes eFigure 3. Inverse-Variance Random-Effects Forest Plot of Associations Between Gestational Age and Mathematics Outcomes eFigure 4. Inverse-Variance Random-Effects Forest Plot of Associations Between Assessment Age and Reading Outcomes eFigure 5. Inverse-Variance Random-Effects Forest Plot of Associations Between Assessment Age and Mathematics Outcomes eFigure 6. Inverse-Variance Random-Effects Forest Plot of Associations Between Birth Era and Reading Outcomes eFigure 7. Inverse-Variance Random-Effects Forest Plot of Associations Between Birth Era and Mathematics Outcomes [file jamanetwopen-3-e202027-s001.pdf]

Supplementary Online Content

McBryde M, Fitzallen GC, Liley HG, Taylor HG, Bora S. Academic outcomes of school-aged children born preterm: A systematic review and meta-analysis. *JAMA Netw Open*. 2020;3(4):e202027. doi:10.1001/jamanetworkopen.2020.2027

eTable 1. List of Keywords Used in Database Searches

eTable 2. List of Assessment Measures from Included Studies

eTable 3. Jackknife Sensitivity Analysis for Academic Subskills Comparisons

eFigure 1. PRISMA Flowchart of Study Selection Process

eFigure 2. Inverse-Variance Random-Effects Forest Plot of Gestational Age and Reading Outcomes for Preterm and Term-Born Children

eFigure 3. Inverse-Variance Random-Effects Forest Plot of Gestational Age and Mathematics Outcomes for Preterm and Term-Born Children

eFigure 4. Inverse-Variance Random-Effects Forest Plot of Assessment Age and Reading Outcomes for Preterm and Term-Born Children

eFigure 5. Inverse-Variance Random-Effects Forest Plot of Assessment Age and Mathematics Outcomes for Preterm and Term-Born Children

eFigure 6. Inverse-Variance Random-Effects Forest Plot of Birth Era and Reading Outcomes for Preterm and Term-Born Children

eFigure 7. Inverse-Variance Random-Effects Forest Plot of Birth Era and Mathematics Outcomes for Preterm and Term-Born Children

This supplementary material has been provided by the authors to give readers additional information about their work.

©2020 McBryde M et al. *JAMA Network Open*.

eTable 1. List of Keywords Used in Database Searches

Population	Exposure	Outcome
child	prematur*	follow up
children	preterm birth	neurodevelopment*
adolescent	“very preterm”	intelligence
“pre-school”	“extremely preterm”	academic
“primary school”	birth weight	“school performance”
“elementary school”	“low birth weight”	delay*
Elementary	“very low birth weight”	deficit*
pre-school*	“extremely low birth weight”	impairment
kindergarten*	“late preterm”	development*
“grade school”	“early term”	reading
school-age*		math*
“secondary school”		spelling
		arithmetic
		numeracy
		literacy
		learning dis*
		developmental dis*
		education*
		decoding
		comprehension
		phonological
		language
		learning
		achievement

©2020 McBryde M et al. *JAMA Network Open*.

eTable 2. List of Assessment Measures from Included Studies

Study	Assessment Tool (cluster or subtest)
Anderson et al, 2003	Wide Range Achievement Test, 3rd Edition (reading[a]; arithmetic[b])
Andreias et al, 2010	Woodcock-Johnson Tests of Achievement (letter-word identification[a]; calculation[c])
Assel et al, 2003	Woodcock-Johnson Tests of Achievement-R (calculation[c])
Botting et al, 1998	Wechsler Objective Reading Dimensions (reading[a])
Bowen et al, 2002	Woodcock Reading Mastery Tests-Revised (total reading[d]; basic reading skills[e]; reading comprehension[f]) TEMA-2 (maths quotient[b])
Brumbaugh et al, 2016	Wide Range Achievement Test, 4th Edition (reading[d]; arithmetic[b])
Chaudhari et al, 2004	Woodcock Reading Mastery Tests-Revised (arithmetic[b])
Cheong et al, 2017	Wide Range Achievement Test, 3rd Edition (reading[a]; arithmetic[b])
Downie et al, 2007	Woodcock Reading Mastery Tests-Revised (word identification[a]; word attack[g])
Doyle et al, 2000	Wide Range Achievement Test, 4th Edition (word reading[a]; mathematical computation[b])
Frye et al, 2009	Woodcock-Johnson Test of Achievement (word attack[g])
Gross et al, 2001	Wechsler Individual Achievement Test (reading[d]; maths composite[h])
Grunau et al., 2002	Wide Range Achievement Test-Revised (word reading[a]; arithmetic[b])
Grunau et al, 2004	Wide Range Achievement Test, 3rd Edition (reading[a]; arithmetic[b])
Hutchinson et al, 2013	Wide Range Achievement Test, 3rd Edition (reading[a]; arithmetic[b])

©2020 McBryde M et al. *JAMA Network Open*.

Study	Assessment Tool (cluster or subtest)
Johnson et al, 2011	Wechsler Individual Achievement Test II (reading composite[d]; word reading[a]; pseudoword decoding[g]; reading comprehension[f]; maths composite[h]; numerical operations[c]; mathematical reasoning[i])
Lee et al, 2011	Woodcock-Johnson III (basic reading skills[e]; passage comprehension[f])
Litt et al, 2012	Woodcock-Johnson III Tests of Achievement (letter-word identification[a]; calculation[c])
Loe et al, 2012	Woodcock-Johnson III Tests of Achievement (broad reading[d])
McGrath et al, 2002	Wide Range Achievement Test, 3rd Edition (reading[a]; arithmetic[b])
Northam et al, 2012	Wechsler Objective Reading Dimensions (reading[a])
Pritchard et al, 2009	Woodcock-Johnson III Tests of Achievement (passage comprehension[f])
Rickards et al, 2001	Wide Range Achievement Test, 3rd Edition (reading[a]; arithmetic[b])
Rose et al, 2011	Woodcock-Johnson III Tests of Achievement (letter-word identification[a], math fluency[j]; applied problems[i])
Sayeur et al, 2015	Wechsler Individual Achievement Test-II (word reading[a])
Short et al, 2003	Woodcock-Johnson Tests of Achievement-Revised (letter-word identification[a]; passage comprehension[f]; calculation[c]; applied problems[i])
Simms et al, 2015	Wechsler Individual Achievement Test-II (maths composite[h])
Tandon et al, 2000	Wide Range Achievement Test-Revised (reading[a]; arithmetic[b])
Taylor et al, 1995	Woodcock-Johnson Tests of Achievement-Revised (word identification[a]; calculation[c]; applied problems[i])
Taylor et al, 2008	Woodcock-Johnson Revised Tests of Cognitive Ability (word identification[a]; passage comprehension[f])

Study	Assessment Tool (cluster or subtest)
Taylor et al, 2011	Woodcock-Johnson Tests of Achievement (letter-word identification[a]; calculation[c]; applied problems[i])
Taylor et al, 2016	Wide Range Achievement Test, 4th Edition (reading[a]; mathematical computation[b])
Woodward et al, 2017	Woodcock-Johnson III Tests of Achievement (math fluency[j])

Note. Clusters and subtests with similar content and thought to test similar academic constructs were compiled to form the following for the purpose of meta-analysis: [a]Word Identification; [b]Mathematical Knowledge; [c]Calculation; [d]Aggregate Measures of Reading; [e]Decoding; [f]Reading Comprehension; [g]Phonological Decoding; [h]Aggregate Measures of Mathematics; [i]Applied Problems; [j]Mathematical Fluency.

©2020 McBryde M et al. *JAMA Network Open*.

eTable 3. Jackknife Sensitivity Analysis for Academic Subskills Comparisons

	**Study removed**	***I*²**	**Subtotal mean difference**	**95% CI**	**p**
Aggregate measures of reading	None	92%	-7.98	-13.05 to -2.91	.002
	Loe et al, 2012	94%	-8.37	-14.35 to -2.39	.006
	Botting et al, 1998	93%	-8.51	-15.26 to -1.76	.01
	Bowen et al, 2002	93%	-7.11	-12.72 to -1.50	.01
	Brumbaugh et al, 2016	93%	-9.06	-14.70 to -3.41	.002
	Gross et al, 2001	92%	-8.99	-15.04 to -2.94	.004
	Johnson et al, 2011	63%	-5.52	-8.22 to -2.81	<.001
Word identification	None	69%	-7.44	-9.08 to -5.80	<.001
	Taylor et al, 2016	70%	-7.33	-9.04 to -5.63	<.001
	Short et al, 2003	70%	-7.56	-9.24 to -5.87	<.001
	Sayeur et al, 2015	70%	-7.50	-9.15 to -5.85	<.001
	Northam et al, 2012	70%	-7.38	-9.07 to -5.68	<.001
	McGrath et al, 2002	70%	-7.46	-9.15 to -5.77	<.001
	Litt et al, 2012	70%	-7.46	-9.18 to -5.74	<.001
	Hutchinson et al, 2013	70%	-7.43	-9.18 to -.5.68	<.001
	Grunau et al, 2004	70%	-7.45	-9.17 to -5.74	<.001
	Doyle et al, 2000	70%	-7.51	-9.26 to -5.77	<.001
	Downie et al, 2007	70%	-7.37	-9.08 to -5.67	<.001
	Andreias et al, 2010	70%	-7.52	-9.25 to -5.78	<.001
	Anderson et al, 2003	70%	-7.48	-9.24 to -5.72	<.001
	Tandon et al, 2000	69%	-7.07	-8.73 to -5.41	<.001
	Rose et al, 2011	69%	-7.66	-9.32 to -5.99	<.001
	Rickards et al, 2001	69%	-7.61	-9.29 to -5.94	<.001
	Grunau et al, 2002	69%	-7.26	-8.92 to -5.59	<.001
	Taylor et al, 2008	68%	-7.69	-9.33 to -6.05	<.001
	Taylor et al, 1995	68%	-7.65	-9.32 to -5.99	<.001
	Taylor et al, 2011	67%	-7.71	-9.34 to -6.08	<.001
	Johnson et al, 2011	63%	-7.09	-8.66 to -5.53	<.001
	Cheong et al, 2017	60%	-6.92	-8.48 to -5.36	<.001
	None	99%	-5.37	-27.41 to -16.67	.63

©2020 McBryde M et al. *JAMA Network Open*.

	Study removed	I^2	Subtotal mean difference	95% CI	p
Pseudoword decoding	Downie et al, 2007	99%	-4.86	-34.73 to 25.01	.75
	Frye et al, 2009	98%	-19.02	-42.73 to 4.70	.12
	Johnson et al, 2011	90%	3.27	-6.91 to 13.45	.53
Reading Comprehension	None	81%	-7.96	-12.15 to -3.76	<.001
	Bowen et al, 2002	84%	-7.48	-12.43 to -2.52	.003
	Lee et al, 2011	84%	-7.66	-12.66 to -2.66	.003
	Short et al, 2003	83%	-8.44	-13.62 to -3.63	<.001
	Pritchard et al, 2009	80%	-8.76	-13.36, -4.16	<.001
	Taylor et al, 2008	79%	-8.91	-13.34 to -4.49	<.001
	Johnson et al, 2011	37%	-6.23	-9.01 to -3.45	<.001
Aggregate measures of mathematics	None	97%	-12.90	-23.38 to -2.43	.02
	Simms et al, 2015	98%	-13.11	-26.33 to 0.12	.05
	Botting et al, 1998	97%	-14.87	-29.70 to -0.04	.05
	Gross et al, 2001	97%	-15.57	-29.15 to -2.00	.02
	Johnson et al, 2011	56%	-7.43	-10.81 to -4.05	<.001
Mathematical knowledge	None	62%	-9.88	-11.68 to -8.08	<.001
	Tandon et al, 2000	65%	-9.52	-11.41 to -7.63	<.001
	Anderson et al, 2003	64%	-10.01	-12.02 to -7.99	<.001
	Bowen et al, 2002	62%	-9.61	-11.46 to -7.77	<.001
	Cheong et al, 2017	54%	-9.38	-11.17 to -7.59	<.001
	Doyle et al, 2000	65%	-9.94	-11.93 to -7.95	<.001
	Grunau et al, 2002	65%	-9.91	-11.83 to -7.99	<.001
	Grunau et al, 2004	62%	-9.63	-11.45 to -7.81	<.001
	Hutchinson et al, 2013	65%	-9.96	-11.93 to -7.99	<.001
	McGrath et al, 2002	65%	-9.87	-11.77 to -7.98	<.001
	Rickards et al, 2001	64%	-10.08	-11.96 to -8.2	<.001
	Taylor et al, 2016	65%	-9.81	-11.74 to -7.89	<.001
	Chaudhari et al, 2004	59%	-10.24	-12.04 to -8.44	<.001
	Brumbaugh et al, 2016	49%	-10.36	-11.93 to -8.78	<.001
Calculation	None	92%	-10.57	-15.62 to -5.52	<.001
	Andreias et al, 2010	93%	-10.85	-17.1 to -4.59	<.001

©2020 McBryde M et al. *JAMA Network Open*.

	Study removed	I^2	Subtotal mean difference	95% CI	p
	Assel et al, 2003	93%	-11.10	-16.84 to -5.36	<.001
	Litt et al, 2012	93%	-10.35	-16.2 to -4.51	<.001
	Short et al, 2003	93%	-10.29	-15.96 to -4.62	<.001
	Taylor et al, 1995	93%	-10.99	-16.85 to -5.13	<.001
	Taylor et al, 2011	89%	-11.89	-16.87 to -6.92	<.001
	Johnson et al, 2011	68%	-8.34	-11.22 to -5.45	<.001
Applied problems	None	91%	-11.41	-17.57 to -5.26	<.001
	Taylor et al, 1995	93%	-11.90	-19.44 to -4.36	.002
	Taylor et al, 2011	93%	-11.48	-19.33 to -3.63	.004
	Short et al, 2003	92%	-12.13	-19.44 to -4.81	.001
	Rose et al, 2011	91%	-12.71	-19.49 to -5.92	<.001
	Johnson et al, 2011	0%	-8.82	-11.07 to -6.57	<.001

Note. No sensitivity analyses are provided for the subgroups of Decoding and Mathematical Fluency as these comparisons included data from two studies only.

©2020 McBryde M et al. *JAMA Network Open*.

eFigure 1. PRISMA Flowchart of Study Selection Process

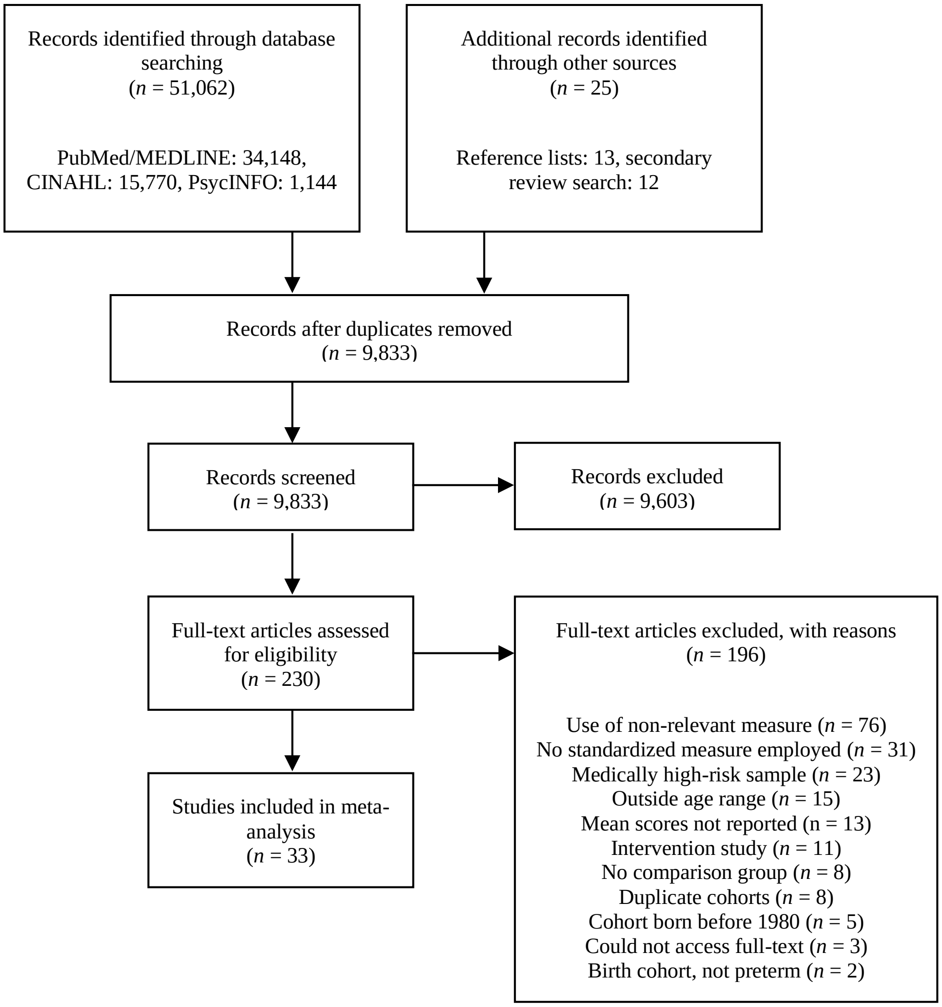

©2020 McBryde M et al. *JAMA Network Open*.

eFigure 2. Inverse-Variance Random-Effects Forest Plot of Gestational Age and Reading Outcomes for Preterm and Term-Born Children

	Preterm			Term				Mean Difference
**Study or Subgroup**	**Mean**	**SD**	**Total**	**Mean**	**SD**	**Total**	**Weight**	**IV, Random, 95% CI**
**4.1.1 < 28 weeks**								
Anderson et al., 2003	96.6	16	259	103.3	14.7	219	6.6%	-6.70 [-9.45, -3.95]
Andreias et al., 2010	90	16	183	96	14	176	6.3%	-6.00 [-9.11, -2.89]
Botting et al., 1998	91.4	11.1	138	96.9	9.49	163	6.8%	-5.50 [-7.86, -3.14]
Bowen et al., 2002	96	15.6	48	108.8	12.1	48	4.7%	-12.80 [-18.39, -7.21]
Cheong et al., 2017	97.1	16.9	133	105.5	13.8	168	6.1%	-8.40 [-11.95, -4.85]
Cheong et al., 2017	94.1	17.1	140	109.4	14.2	189	6.1%	-15.30 [-18.78, -11.82]
Downie et al., 2007	95.17	10.67	39	104	7.3	15	5.1%	-8.83 [-13.82, -3.84]
Doyle et al., 2000	95.1	14.1	223	101.2	14.3	160	6.5%	-6.10 [-8.99, -3.21]
Grunau et al., 2002	94.5	16.5	74	107	14.1	30	4.3%	-12.50 [-18.79, -6.21]
Grunau et al., 2004	103.54	10.85	53	110.59	9.85	31	5.4%	-7.05 [-11.58, -2.52]
Hutchinson et al., 2013	98	16.1	189	105.5	13.8	173	6.4%	-7.50 [-10.58, -4.42]
Johnson et al., 2011	80.2	20.3	212	98.5	11.6	153	6.2%	-18.30 [-21.59, -15.01]
Litt et al., 2012	88.6	21.9	181	95.5	14.1	115	5.7%	-6.90 [-11.00, -2.80]
McGrath et al., 2002	90.8	22.3	48	100.1	19.6	37	3.0%	-9.30 [-18.23, -0.37]
Northam et al., 2012	96	14	50	105	12	30	4.6%	-9.00 [-14.79, -3.21]
Pritchard et al., 2009	108.88	15.6	102	113	15.5	108	5.6%	-4.12 [-8.33, 0.09]
Taylor et al., 2011	106.07	13.47	194	107.9	16.9	70	5.5%	-1.83 [-6.22, 2.56]
Taylor et al., 2016	98.2	19.6	194	107.9	16.9	70	5.2%	-9.70 [-14.52, -4.88]
**Subtotal (95% CI)**			**2460**			**1955**	**100.0%**	**-8.54 [-10.52, -6.55]**
Heterogeneity: Tau² = 13.65; Chi² = 79.76, df = 17 (P < 0.00001); I² = 79%								
Test for overall effect: Z = 8.43 (P < 0.00001)								
**4.1.2 28 - 32 weeks**								
Frye et al., 2009	102.74	19.17	94	93.53	17.29	97	10.8%	9.21 [4.03, 14.39]
Frye et al., 2009	100.97	20.77	62	93.53	17.29	97	9.6%	7.44 [1.23, 13.65]
Gross et al., 2001	86.75	10	118	90	10.25	119	13.8%	-3.25 [-5.83, -0.67]
Loe et al., 2012	105	13.6	72	111	10.09	42	11.7%	-6.00 [-10.38, -1.62]
McGrath et al., 2002	95	22.8	52	100.1	19.6	37	6.9%	-5.10 [-13.95, 3.75]
Rickards et al., 2001	96.8	14.4	120	100.4	12.7	41	11.4%	-3.60 [-8.26, 1.06]
Rose et al., 2011	97.95	14.54	44	100.6	9.76	86	11.3%	-2.65 [-7.42, 2.12]
Sayeur et al., 2015	106	23.1	10	105	18.2	10	2.5%	1.00 [-17.23, 19.23]
Short et al., 2003	102.3	17	75	107.6	18	99	10.7%	-5.30 [-10.53, -0.07]
Taylor et al., 2008	98.8	21	155	102	15.4	82	11.4%	-3.20 [-7.89, 1.49]
**Subtotal (95% CI)**			**802**			**710**	**100.0%**	**-1.42 [-4.58, 1.75]**
Heterogeneity: Tau² = 17.18; Chi² = 33.82, df = 9 (P < 0.0001); I² = 73%								
Test for overall effect: Z = 0.88 (P = 0.38)								
**4.1.3 33 - 37 weeks**								
Brumbaugh et al., 2016	98.8	15	52	100.9	15	74	27.6%	-2.10 [-7.42, 3.22]
McGrath et al., 2002	94.6	20.1	51	100.1	19.6	37	21.2%	-5.50 [-13.89, 2.89]
Tandon et al., 2000	108	14.7	27	117.3	11.1	28	24.3%	-9.30 [-16.20, -2.40]
Tandon et al., 2000	96	12.2	32	111.1	10.3	29	26.9%	-15.10 [-20.75, -9.45]
**Subtotal (95% CI)**			**162**			**168**	**100.0%**	**-8.07 [-14.29, -1.84]**
Heterogeneity: Tau² = 29.21; Chi² = 11.27, df = 3 (P = 0.01); I² = 73%								
Test for overall effect: Z = 2.54 (P = 0.01)								

Mean Difference
IV, Random, 95% CI

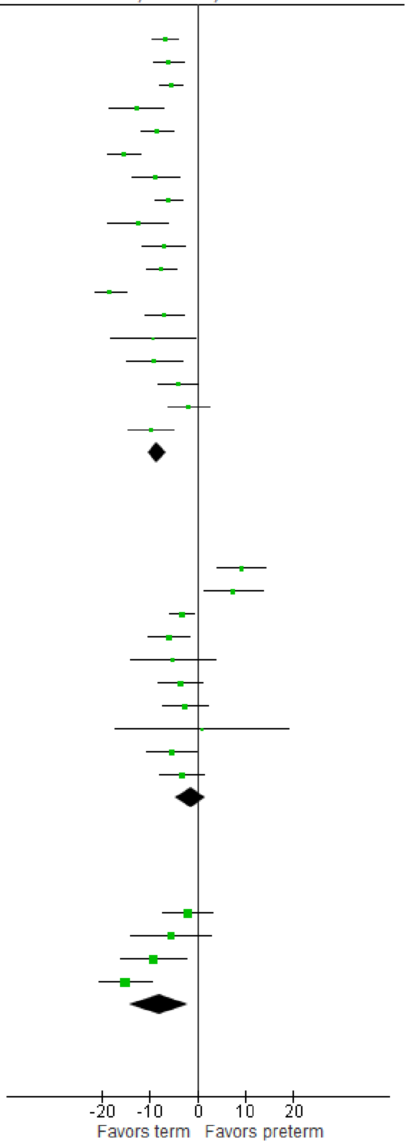

©2020 McBryde M et al. *JAMA Network Open*.

eFigure 3. Inverse-Variance Random-Effects Forest Plot of Gestational Age and Mathematics Outcomes for Preterm and Term-Born Children

	Preterm			Term				Mean Difference
**Study or Subgroup**	**Mean**	**SD**	**Total**	**Mean**	**SD**	**Total**	**Weight**	**IV, Random, 95% CI**
**5.1.1 < 28 weeks**								
Andreias et al., 2010	89	15	183	98	14	176	7.3%	-9.00 [-12.00, -6.00]
Botting et al., 1998	96.3	13.2	102	103.4	9	108	7.3%	-7.10 [-10.17, -4.03]
Bowen et al., 2002	90.8	11	48	104.5	12.2	48	6.5%	-13.70 [-18.35, -9.05]
Cheong et al., 2017	89.9	17.5	131	99	14.5	168	7.0%	-9.10 [-12.81, -5.39]
Cheong et al., 2017	89.4	18.9	140	105.1	13.4	188	7.0%	-15.70 [-19.37, -12.03]
Doyle et al., 2000	85.4	14.7	223	94.8	15.1	160	7.3%	-9.40 [-12.43, -6.37]
Grunau et al., 2002	90.3	11	74	99.9	10.5	30	6.5%	-9.60 [-14.12, -5.08]
Grunau et al., 2004	91.35	14.25	53	106.29	14.45	31	5.5%	-14.94 [-21.31, -8.57]
Hutchinson et al., 2013	90	16.9	189	99.1	14.5	173	7.2%	-9.10 [-12.34, -5.86]
Johnson et al., 2011	71.2	20.9	215	98.5	15	153	7.0%	-27.30 [-30.97, -23.63]
Litt et al., 2012	81.3	20.7	181	93.2	17.2	115	6.6%	-11.90 [-16.26, -7.54]
McGrath et al., 2002	88.7	19.8	48	100.2	14.8	37	5.0%	-11.50 [-18.86, -4.14]
Taylor et al., 1995	92.95	14.8	88	102.3	12.7	58	6.5%	-9.35 [-13.85, -4.85]
Taylor et al., 2011	95.1	17.04	140	106.12	16.96	111	6.7%	-11.02 [-15.25, -6.79]
Taylor et al., 2016	88.8	18.4	194	99.7	14.1	70	6.7%	-10.90 [-15.10, -6.70]
**Subtotal (95% CI)**			**2009**			**1626**	**100.0%**	**-11.92 [-14.60, -9.24]**
Heterogeneity: Tau² = 23.30; Chi² = 96.10, df = 14 (P < 0.00001); I² = 85%								
Test for overall effect: Z = 8.72 (P < 0.00001)								
**5.1.2 28 - 32 weeks**								
Assel et al., 2003	93.7	22.3	160	101	15.4	90	12.2%	-7.30 [-12.00, -2.60]
Gross et al., 2001	104	9	48	109	10	83	24.1%	-5.00 [-8.33, -1.67]
McGrath et al., 2002	90.3	19	52	100.2	14.8	37	5.5%	-9.90 [-16.93, -2.87]
Rickards et al., 2001	89	13.8	120	95.9	13.6	41	11.5%	-6.90 [-11.74, -2.06]
Rose et al., 2011	97.5	13	42	103.69	10	84	13.5%	-6.19 [-10.67, -1.71]
Short et al., 2003	103.8	17	75	112.2	15	99	11.5%	-8.40 [-13.25, -3.55]
Simms et al., 2015	91.29	18.81	113	103.56	20.69	77	8.1%	-12.27 [-18.05, -6.49]
Woodwood et al., 2017	89	17	100	99.1	15.5	107	13.7%	-10.10 [-14.54, -5.66]
**Subtotal (95% CI)**			**710**			**618**	**100.0%**	**-7.60 [-9.25, -5.96]**
Heterogeneity: Tau² = 0.05; Chi² = 7.06, df = 7 (P = 0.42); I² = 1%								
Test for overall effect: Z = 9.04 (P < 0.00001)								
**5.1.3 33 - 37 weeks**								
Brumbaugh et al., 2016	98.05	15	52	98.65	15	74	21.0%	-0.60 [-5.92, 4.72]
Chaudhari et al., 2004	82.7	16.9	180	87.8	15.8	90	23.4%	-5.10 [-9.19, -1.01]
McGrath et al., 2002	91.3	13.9	51	100.2	14.8	37	19.4%	-8.90 [-15.01, -2.79]
Tandon et al., 2000	100.2	13.8	32	115.2	13.3	29	18.1%	-15.00 [-21.80, -8.20]
Tandon et al., 2000	113.4	14.9	27	125.7	10.4	28	18.1%	-12.30 [-19.11, -5.49]
**Subtotal (95% CI)**			**342**			**258**	**100.0%**	**-7.98 [-12.81, -3.16]**
Heterogeneity: Tau² = 21.51; Chi² = 14.39, df = 4 (P = 0.006); I² = 72%								
Test for overall effect: Z = 3.24 (P = 0.001)								

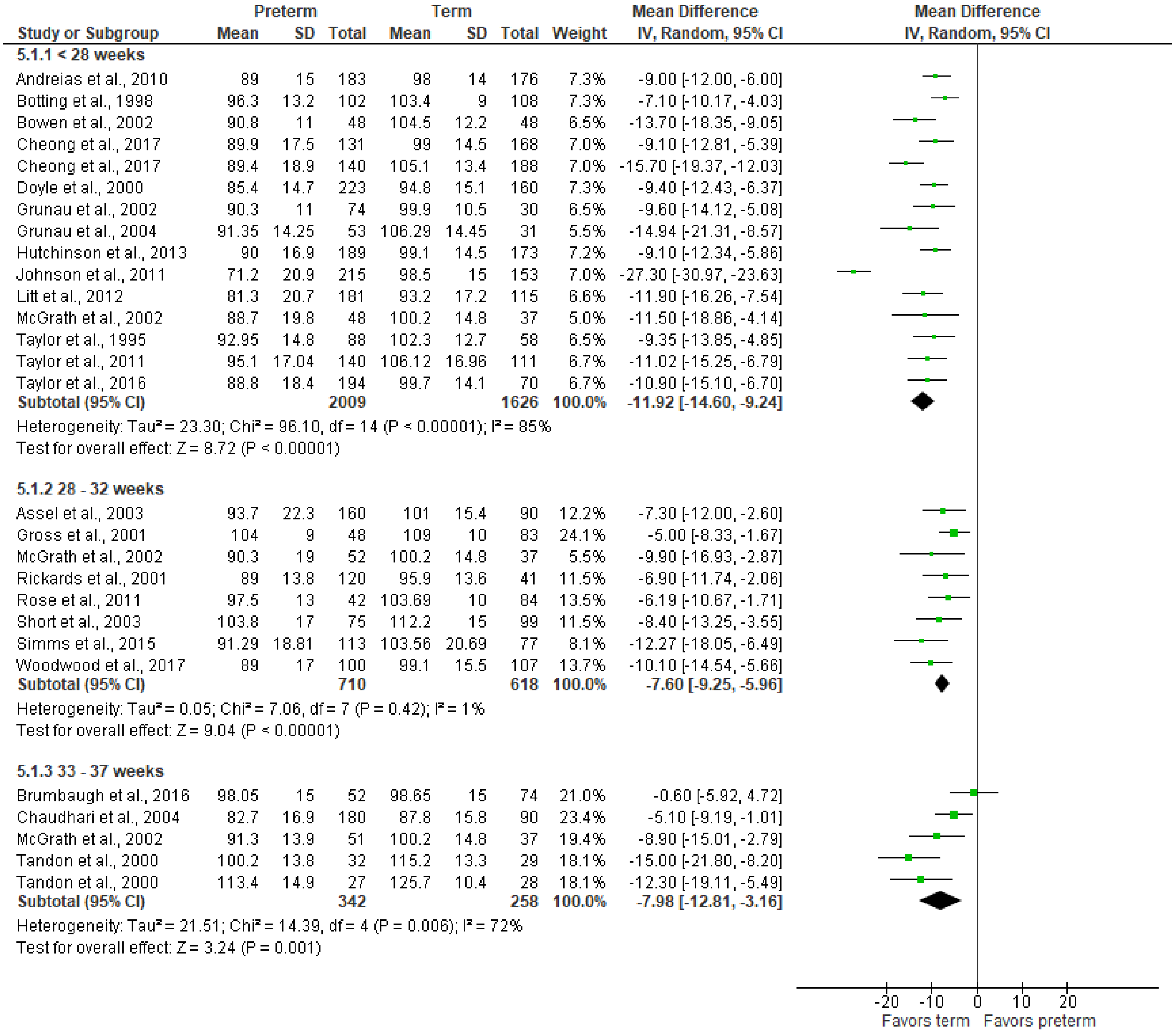

©2020 McBryde M et al. *JAMA Network Open*.

eFigure 4. Inverse-Variance Random-Effects Forest Plot of Assessment Age and Reading Outcomes for Preterm and Term-Born Children

Study or Subgroup	Preterm Mean	Preterm SD	Preterm Total	Term Mean	Term SD	Term Total	Weight	Mean Difference IV, Random, 95% CI
**6.1.1 Ages 5 - 8**								
Andreias et al., 2010	90	16	183	96	14	176	10.0%	-6.00 [-9.11, -2.89]
Bowen et al., 2002	96	15.6	48	108.8	12.1	48	7.1%	-12.80 [-18.39, -7.21]
Cheong et al., 2017	97.1	16.9	133	105.5	13.8	168	9.5%	-8.40 [-11.95, -4.85]
Cheong et al., 2017	94.1	17.1	140	109.4	14.2	189	9.6%	-15.30 [-18.78, -11.82]
Hutchinson et al., 2013	98	16.1	189	105.5	13.8	173	10.1%	-7.50 [-10.58, -4.42]
McGrath et al., 2002	93.47	21.73	151	100.1	19.6	37	5.6%	-6.63 [-13.83, 0.57]
Pritchard et al., 2009	108.88	15.6	102	113	15.5	108	8.7%	-4.12 [-8.33, 0.09]
Sayeur et al., 2015	106	23.1	10	105	18.2	10	1.4%	1.00 [-17.23, 19.23]
Short et al., 2003	102.3	17	75	107.6	18	99	7.5%	-5.30 [-10.53, -0.07]
Tandon et al., 2000	108	14.7	27	117.3	11.1	28	5.9%	-9.30 [-16.20, -2.40]
Taylor et al., 2008	98.8	21	155	102	15.4	82	8.1%	-3.20 [-7.89, 1.49]
Taylor et al., 2011	106.07	13.47	194	107.9	16.9	70	8.5%	-1.83 [-6.22, 2.56]
Taylor et al., 2016	98.2	19.6	194	107.9	16.9	70	8.0%	-9.70 [-14.52, -4.88]
**Subtotal (95% CI)**			**1601**			**1258**	**100.0%**	**-7.38 [-9.69, -5.07]**
Heterogeneity: Tau^2 = 11.38; Chi^2 = 38.60, df = 12 (P = 0.0001); I^2 = 69%								
Test for overall effect: Z = 6.26 (P < 0.00001)								
**6.1.2 Ages 9 - 11**								
Brumbaugh et al., 2016	98.8	15	52	100.9	15	74	13.9%	-2.10 [-7.42, 3.22]
Downie et al., 2007	95.17	10.67	39	104	7.3	15	14.2%	-8.83 [-13.82, -3.84]
Gross et al., 2001	86.75	10	118	90	10.25	119	15.5%	-3.25 [-5.83, -0.67]
Grunau et al., 2002	94.5	16.5	74	107	14.1	30	13.2%	-12.50 [-18.79, -6.21]
Johnson et al., 2011	80.2	20.3	212	98.5	11.6	153	15.2%	-18.30 [-21.59, -15.01]
Rose et al., 2011	97.95	14.54	44	100.6	9.76	86	14.3%	-2.65 [-7.42, 2.12]
Tandon et al., 2000	96	12.2	32	111.1	10.3	29	13.7%	-15.10 [-20.75, -9.45]
**Subtotal (95% CI)**			**571**			**506**	**100.0%**	**-8.93 [-14.42, -3.43]**
Heterogeneity: Tau^2 = 49.10; Chi^2 = 68.08, df = 6 (P < 0.00001); I^2 = 91%								
Test for overall effect: Z = 3.18 (P = 0.001)								
**6.1.3 Ages 12 - 18**								
Botting et al., 1998	91.4	11.1	138	96.9	9.49	163	13.0%	-5.50 [-7.86, -3.14]
Doyle et al., 2000	95.1	14.1	223	101.2	14.3	160	12.6%	-6.10 [-8.99, -3.21]
Frye et al., 2009	100.97	20.77	62	93.53	17.29	97	9.4%	7.44 [1.23, 13.65]
Frye et al., 2009	102.74	19.17	94	93.53	17.29	97	10.4%	9.21 [4.03, 14.39]
Grunau et al., 2004	103.54	10.85	53	110.59	9.85	31	11.1%	-7.05 [-11.58, -2.52]
Litt et al., 2012	88.6	21.9	181	95.5	14.1	115	11.5%	-6.90 [-11.00, -2.80]
Loe et al., 2012	105	13.6	72	111	10.09	42	11.2%	-6.00 [-10.38, -1.62]
Northam et al., 2012	96	14	50	105	12	30	9.8%	-9.00 [-14.79, -3.21]
Rickards et al., 2001	96.8	14.4	120	100.4	12.7	41	10.9%	-3.60 [-8.26, 1.06]
**Subtotal (95% CI)**			**993**			**776**	**100.0%**	**-3.35 [-6.70, 0.01]**
Heterogeneity: Tau^2 = 21.10; Chi^2 = 48.40, df = 8 (P < 0.00001); I^2 = 83%								
Test for overall effect: Z = 1.96 (P = 0.05)								

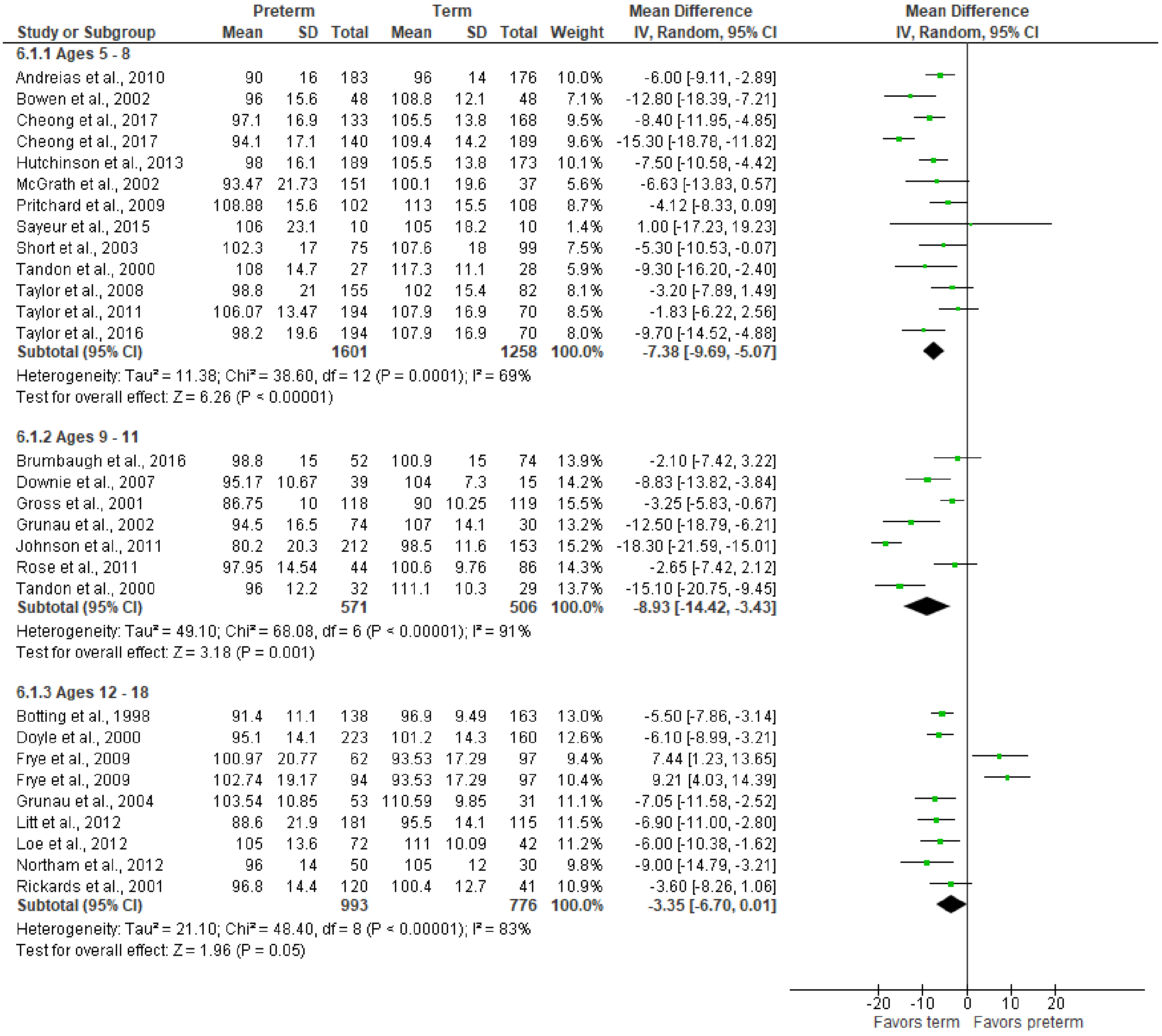

©2020 McBryde M et al. *JAMA Network Open*.

eFigure 5. Inverse-Variance Random-Effects Forest Plot of Assessment Age and Mathematics Outcomes for Preterm and Term-Born Children

Study or Subgroup	Preterm Mean	Preterm SD	Preterm Total	Term Mean	Term SD	Term Total	Weight	Mean Difference IV, Random, 95% CI
**7.1.1 Ages 5 - 8**								
Andreias et al., 2010	89	15	183	98	14	176	13.2%	-9.00 [-12.00, -6.00]
Assel et al., 2003	93.7	22.3	160	101	15.4	90	7.1%	-7.30 [-12.00, -2.60]
Bowen et al., 2002	90.8	11	48	104.5	12.2	48	7.2%	-13.70 [-18.35, -9.05]
Cheong et al., 2017	89.9	17.5	131	99	14.5	168	10.0%	-9.10 [-12.81, -5.39]
Cheong et al., 2017	89.4	18.9	140	105.1	13.4	188	10.2%	-15.70 [-19.37, -12.03]
Hutchinson et al., 2013	90	16.9	189	99.1	14.5	173	12.0%	-9.10 [-12.34, -5.86]
McGrath et al., 2002	90.1	17.57	151	100.2	14.8	37	5.4%	-10.10 [-15.63, -4.57]
Short et al., 2003	103.8	17	75	112.2	15	99	6.7%	-8.40 [-13.25, -3.55]
Tandon et al., 2000	113.4	14.9	27	125.7	10.4	28	3.8%	-12.30 [-19.11, -5.49]
Taylor et al., 1995	92.95	14.8	88	102.3	12.7	58	7.6%	-9.35 [-13.85, -4.85]
Taylor et al., 2011	95.1	17.04	140	106.12	16.96	111	8.3%	-11.02 [-15.25, -6.79]
Taylor et al., 2016	88.8	18.4	194	99.7	14.1	70	8.4%	-10.90 [-15.10, -6.70]
**Subtotal (95% CI)**			**1526**			**1246**	**100.0%**	**-10.42 [-11.83, -9.01]**
Heterogeneity: Tau² = 1.58; Chi² = 14.86, df = 11 (P = 0.19); I² = 26%								
Test for overall effect: Z = 14.47 (P < 0.00001)								
**7.1.2 Ages 9 - 11**								
Brumbaugh et al., 2016	98.05	15	52	98.65	15	74	12.3%	-0.60 [-5.92, 4.72]
Gross et al., 2001	104	9	48	109	10	83	13.0%	-5.00 [-8.33, -1.67]
Grunau et al., 2002	90.3	11	74	99.9	10.5	30	12.6%	-9.60 [-14.12, -5.08]
Johnson et al., 2011	71.2	20.9	215	98.5	15	153	12.9%	-27.30 [-30.97, -23.63]
Rose et al., 2011	97.5	13	42	103.69	10	84	12.6%	-6.19 [-10.67, -1.71]
Simms et al., 2015	91.29	18.81	113	103.56	20.69	77	12.1%	-12.27 [-18.05, -6.49]
Tandon et al., 2000	100.2	13.8	32	115.2	13.3	29	11.7%	-15.00 [-21.80, -8.20]
Woodwood et al., 2017	89	17	100	99.1	15.5	107	12.7%	-10.10 [-14.54, -5.66]
**Subtotal (95% CI)**			**676**			**637**	**100.0%**	**-10.76 [-17.12, -4.41]**
Heterogeneity: Tau² = 78.02; Chi² = 109.41, df = 7 (P < 0.00001); I² = 94%								
Test for overall effect: Z = 3.32 (P = 0.0009)								
**7.1.3 Ages 12 - 18**								
Botting et al., 1998	96.3	13.2	102	103.4	9	108	21.5%	-7.10 [-10.17, -4.03]
Chaudhari et al., 2004	82.7	16.9	180	87.8	15.8	90	16.9%	-5.10 [-9.19, -1.01]
Doyle et al., 2000	85.4	14.7	223	94.8	15.1	160	21.7%	-9.40 [-12.43, -6.37]
Grunau et al., 2004	91.35	14.25	53	106.29	14.45	31	10.0%	-14.94 [-21.31, -8.57]
Litt et al., 2012	81.3	20.7	181	93.2	17.2	115	15.9%	-11.90 [-16.26, -7.54]
Rickards et al., 2001	89	13.8	120	95.9	13.6	41	14.1%	-6.90 [-11.74, -2.06]
**Subtotal (95% CI)**			**859**			**545**	**100.0%**	**-8.77 [-11.18, -6.37]**
Heterogeneity: Tau² = 4.54; Chi² = 10.47, df = 5 (P = 0.06); I² = 52%								
Test for overall effect: Z = 7.16 (P < 0.00001)								

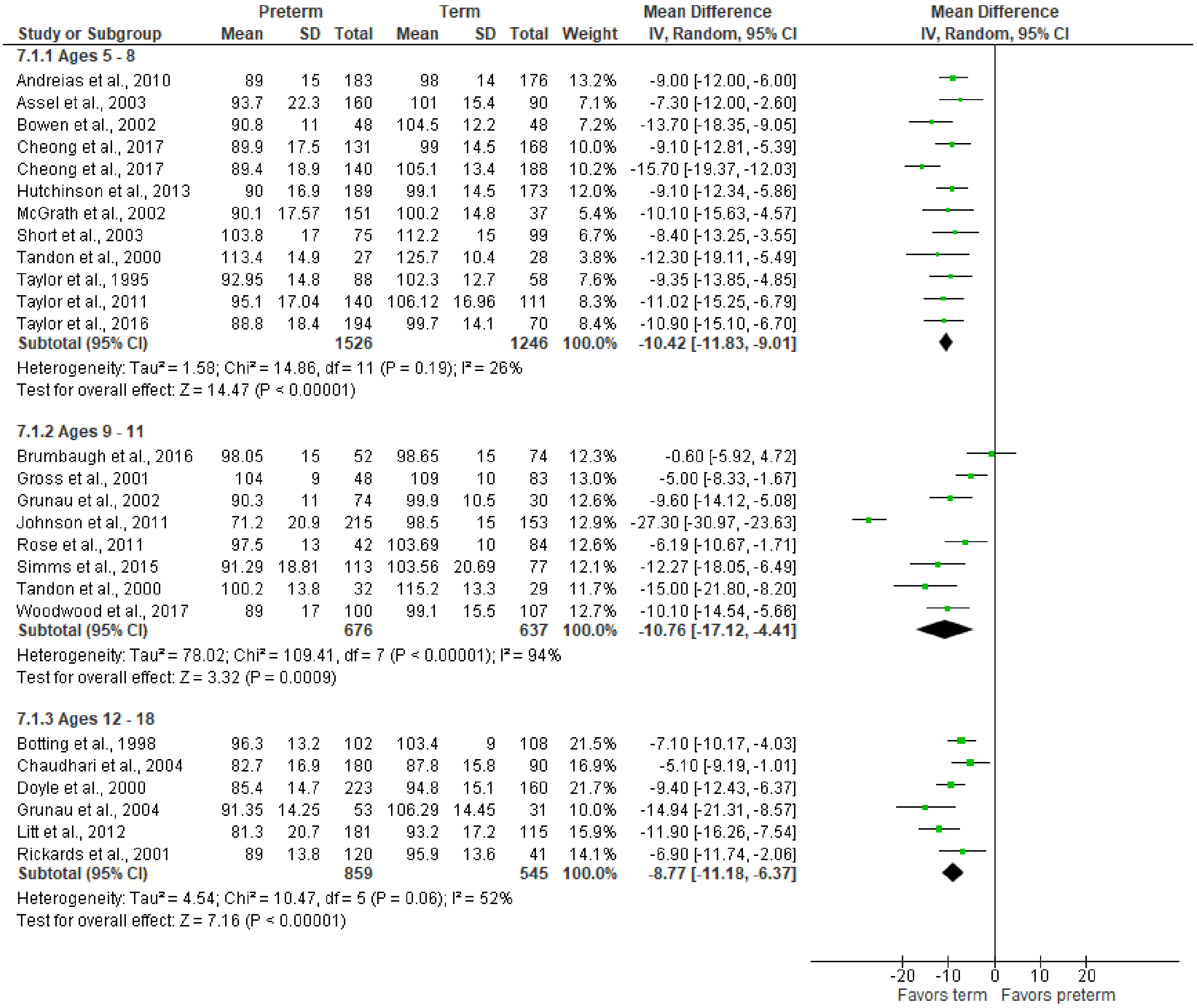

©2020 McBryde M et al. *JAMA Network Open*.

eFigure 6. Inverse-Variance Random-Effects Forest Plot of Birth Era and Reading Outcomes for Preterm and Term-Born Children

	Preterm			Term				Mean Difference
Study or Subgroup	Mean	SD	Total	Mean	SD	Total	Weight	IV, Random, 95% CI
**8.1.1 1980 - 1990**								
Botting et al., 1998	91.4	11.1	138	96.9	9.49	163	11.9%	-5.50 [-7.86, -3.14]
Bowen et al., 2002	96	15.6	48	108.8	12.1	48	6.9%	-12.80 [-18.39, -7.21]
Downie et al., 2007	95.17	10.67	39	104	7.3	15	7.7%	-8.83 [-13.82, -3.84]
Gross et al., 2001	86.75	10	118	90	10.25	119	11.6%	-3.25 [-5.83, -0.67]
Grunau et al., 2002	94.5	16.5	74	107	14.1	30	6.0%	-12.50 [-18.79, -6.21]
Grunau et al., 2004	103.54	10.85	53	110.59	9.85	31	8.3%	-7.05 [-11.58, -2.52]
McGrath et al., 2002	93.47	21.73	151	100.1	19.6	37	5.1%	-6.63 [-13.83, 0.57]
Northam et al., 2012	96	14	50	105	12	30	6.6%	-9.00 [-14.79, -3.21]
Rickards et al., 2001	96.8	14.4	120	100.4	12.7	41	8.1%	-3.60 [-8.26, 1.06]
Short et al., 2003	102.3	17	75	107.6	18	99	7.3%	-5.30 [-10.53, -0.07]
Tandon et al., 2000	96	12.2	32	111.1	10.3	29	6.8%	-15.10 [-20.75, -9.45]
Tandon et al., 2000	108	14.7	27	117.3	11.1	28	5.4%	-9.30 [-16.20, -2.40]
Taylor et al., 2008	98.8	21	155	102	15.4	82	8.1%	-3.20 [-7.89, 1.49]
**Subtotal (95% CI)**			**1080**			**752**	**100.0%**	**-7.34 [-9.38, -5.30]**
Heterogeneity: Tau² = 7.63; Chi² = 29.83, df = 12 (P = 0.003); I² = 60%								
Test for overall effect: Z = 7.05 (P < 0.00001)								
**8.1.2 1991 - 2000**								
Andreias et al., 2010	90	16	183	96	14	176	8.8%	-6.00 [-9.11, -2.89]
Brumbaugh et al., 2016	98.8	15	52	100.9	15	74	7.8%	-2.10 [-7.42, 3.22]
Cheong et al., 2017	97.1	16.9	133	105.5	13.8	168	8.6%	-8.40 [-11.95, -4.85]
Doyle et al., 2000	95.1	14.1	223	101.2	14.3	160	8.9%	-6.10 [-8.99, -3.21]
Frye et al., 2009	100.97	20.77	62	93.53	17.29	97	7.4%	7.44 [1.23, 13.65]
Frye et al., 2009	102.74	19.17	94	93.53	17.29	97	7.9%	9.21 [4.03, 14.39]
Hutchinson et al., 2013	98	16.1	189	105.5	13.8	173	8.8%	-7.50 [-10.58, -4.42]
Johnson et al., 2011	80.2	20.3	212	98.5	11.6	153	8.7%	-18.30 [-21.59, -15.01]
Litt et al., 2012	88.6	21.9	181	95.5	14.1	115	8.4%	-6.90 [-11.00, -2.80]
Loe et al., 2012	105	13.6	72	111	10.09	42	8.3%	-6.00 [-10.38, -1.62]
Pritchard et al., 2009	108.88	15.6	102	113	15.5	108	8.3%	-4.12 [-8.33, 0.09]
Rose et al., 2011	97.95	14.54	44	100.6	9.76	86	8.1%	-2.65 [-7.42, 2.12]
**Subtotal (95% CI)**			**1547**			**1449**	**100.0%**	**-4.58 [-8.18, -0.97]**
Heterogeneity: Tau² = 36.01; Chi² = 111.98, df = 11 (P < 0.00001); I² = 90%								
Test for overall effect: Z = 2.49 (P = 0.01)								
**8.1.3 2001 - 2018**								
Cheong et al., 2017	94.1	17.1	140	109.4	14.2	189	30.5%	-15.30 [-18.78, -11.82]
Sayeur et al., 2015	106	23.1	10	105	18.2	10	11.3%	1.00 [-17.23, 19.23]
Taylor et al., 2011	106.07	13.47	194	107.9	16.9	70	29.4%	-1.83 [-6.22, 2.56]
Taylor et al., 2016	98.2	19.6	194	107.9	16.9	70	28.8%	-9.70 [-14.52, -4.88]
**Subtotal (95% CI)**			**538**			**339**	**100.0%**	**-7.89 [-15.46, -0.32]**
Heterogeneity: Tau² = 45.68; Chi² = 23.60, df = 3 (P < 0.0001); I² = 87%								
Test for overall effect: Z = 2.04 (P = 0.04)								

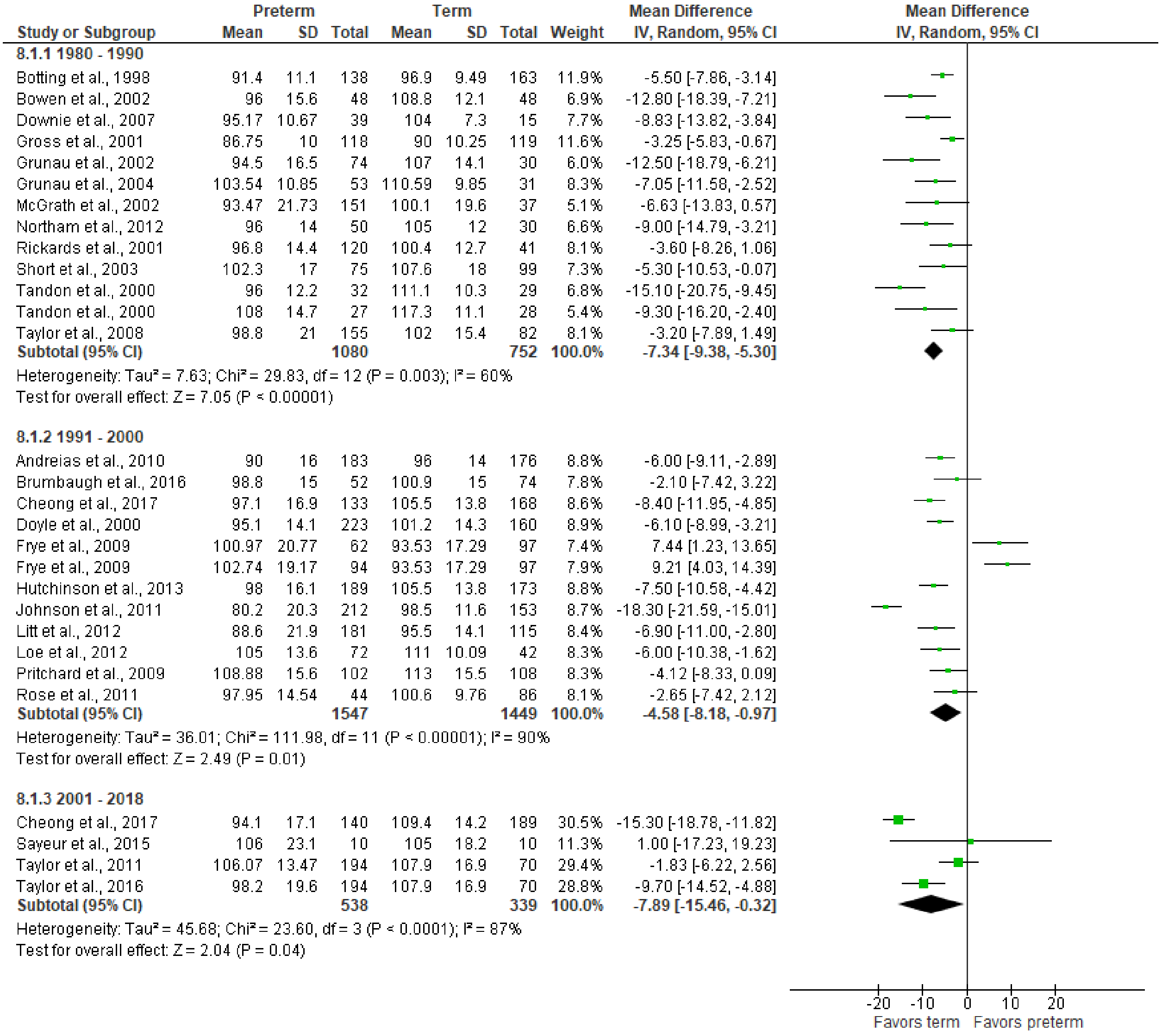

©2020 McBryde M et al. *JAMA Network Open*.

eFigure 7. Inverse-Variance Random-Effects Forest Plot of Birth Era and Mathematics Outcomes for Preterm and Term-Born Children

Study or Subgroup	Preterm Mean	Preterm SD	Preterm Total	Term Mean	Term SD	Term Total	Weight	Mean Difference IV, Random, 95% CI
**9.1.1 1980 - 1990**								
Assel et al., 2003	93.7	22.3	160	101	15.4	90	7.8%	-7.30 [-12.00, -2.60]
Botting et al., 1998	96.3	13.2	102	103.4	9	108	11.4%	-7.10 [-10.17, -4.03]
Bowen et al., 2002	90.8	11	48	104.5	12.2	48	7.9%	-13.70 [-18.35, -9.05]
Chaudhari et al., 2004	82.7	16.9	180	87.8	15.8	90	9.0%	-5.10 [-9.19, -1.01]
Gross et al., 2001	104	9	48	109	10	83	10.7%	-5.00 [-8.33, -1.67]
Grunau et al., 2002	90.3	11	74	99.9	10.5	30	8.2%	-9.60 [-14.12, -5.08]
Grunau et al., 2004	91.35	14.2	53	106.29	14.45	31	5.4%	-14.94 [-21.30, -8.58]
McGrath et al., 2002	90.1	17.57	151	100.2	14.8	37	6.5%	-10.10 [-15.63, -4.57]
Rickards et al., 2001	89	13.8	120	95.9	13.6	41	7.6%	-6.90 [-11.74, -2.06]
Short et al., 2003	103.8	17	75	112.2	15	99	7.6%	-8.40 [-13.25, -3.55]
Tandon et al., 2000	113.4	14.9	27	125.7	10.4	28	4.9%	-12.30 [-19.11, -5.49]
Tandon et al., 2000	100.2	13.8	32	115.2	13.3	29	4.9%	-15.00 [-21.80, -8.20]
Taylor et al., 1995	92.95	14.8	88	102.3	12.7	58	8.2%	-9.35 [-13.85, -4.85]
**Subtotal (95% CI)**			**1158**			**772**	**100.0%**	**-8.96 [-10.74, -7.18]**
Heterogeneity: Tau² = 4.78; Chi² = 22.48, df = 12 (P = 0.03); I² = 47%								
Test for overall effect: Z = 9.87 (P < 0.00001)								
**9.1.2 1991 - 2000**								
Andreias et al., 2010	89	15	183	98	14	176	11.5%	-9.00 [-12.00, -6.00]
Brumbaugh et al., 2016	98.05	15	52	98.65	15	74	10.4%	-0.60 [-5.92, 4.72]
Cheong et al., 2017	89.9	17.5	131	99	14.5	168	11.2%	-9.10 [-12.81, -5.39]
Doyle et al., 2000	85.4	14.7	223	94.8	15.1	160	11.5%	-9.40 [-12.43, -6.37]
Hutchinson et al., 2013	90	16.9	189	99.1	14.5	173	11.4%	-9.10 [-12.34, -5.86]
Johnson et al., 2011	71.2	20.9	215	98.5	15	153	11.3%	-27.30 [-30.97, -23.63]
Litt et al., 2012	81.3	20.7	181	93.2	17.2	115	10.9%	-11.90 [-16.26, -7.54]
Rose et al., 2011	97.5	13	42	103.69	10	84	10.8%	-6.19 [-10.67, -1.71]
Woodwood et al., 2017	89	17	100	99.1	15.5	107	10.9%	-10.10 [-14.54, -5.66]
**Subtotal (95% CI)**			**1316**			**1210**	**100.0%**	**-10.39 [-14.83, -5.94]**
Heterogeneity: Tau² = 42.23; Chi² = 100.34, df = 8 (P < 0.00001); I² = 92%								
Test for overall effect: Z = 4.58 (P < 0.00001)								
**9.1.3 2001 - 2018**								
Cheong et al., 2017	89.4	18.9	140	105.1	13.4	188	32.0%	-15.70 [-19.37, -12.03]
Simms et al., 2015	91.29	18.81	113	103.56	20.69	77	15.7%	-12.27 [-18.05, -6.49]
Taylor et al., 2011	95.1	17.04	140	106.12	16.96	111	26.0%	-11.02 [-15.25, -6.79]
Taylor et al., 2016	88.8	18.4	194	99.7	14.1	70	26.3%	-10.90 [-15.10, -6.70]
**Subtotal (95% CI)**			**587**			**446**	**100.0%**	**-12.68 [-15.16, -10.21]**
Heterogeneity: Tau² = 1.48; Chi² = 3.90, df = 3 (P = 0.27); I² = 23%								
Test for overall effect: Z = 10.04 (P < 0.00001)								

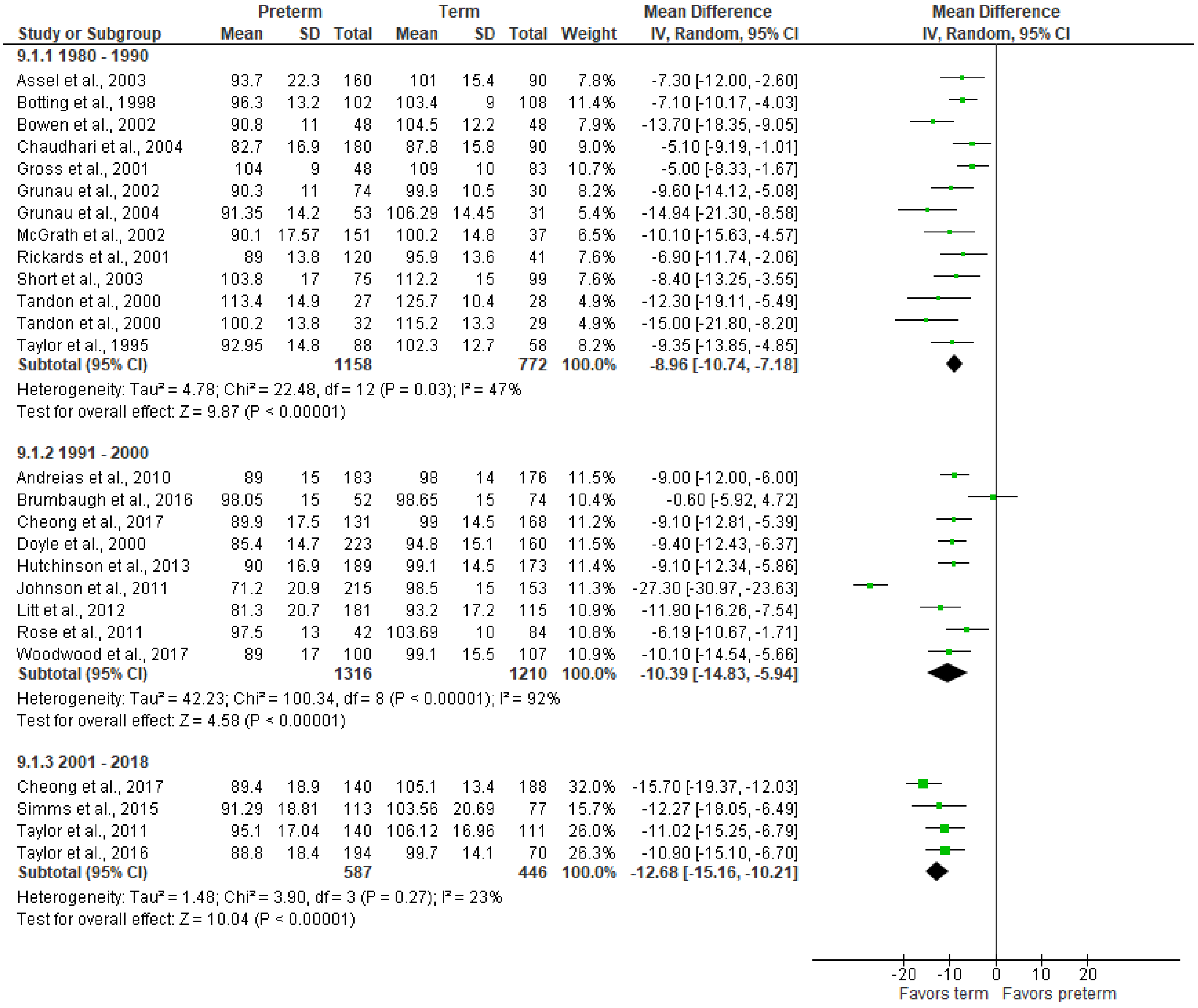

©2020 McBryde M et al. *JAMA Network Open*.
